# Gypenosides ameliorate morphine-induced immunosuppression with an increased proportion of thymic T lymphocyte subsets and are involved in the regulation of the cAMP-CREM/CREB-IL-2 pathway

**DOI:** 10.1016/j.gendis.2023.05.026

**Published:** 2023-07-28

**Authors:** Hui Wang, Zhonghao Li, Qisheng Wang, Weixin Lin, Ziting Zhou, Xinru Mu, Yongwei Jiang, Shengfeng Lu, Shaodong Chen, Zhigang Lu

**Affiliations:** a Jiangsu Collaborative Innovation Center of Traditional Chinese Medicine in Prevention and Treatment of Tumor, Nanjing University of Chinese Medicine, Nanjing, Jiangsu 210023, China; b Key Laboratory of Acupuncture and Medicine Research of Ministry of Education, Nanjing University of Chinese Medicine, Nanjing, Jiangsu 210023, China; c College of Pharmacy, Nanjing University of Chinese Medicine, Nanjing, Jiangsu 210023, China; d School of Medicine, Xiamen University, Xiamen, Fujian 361102, China

Opioid abuse can suppress the lymphatic system function, and produce severe immunosuppression that poses a significant risk of opportunistic infections such as methicillin-resistant *Staphylococcus aureus* (MRSA) pneumonia.^1^^,^^2^ Gypenosides (Gps) are the most important immunomodulator components in the Chinese herbal medicine *Gynostemma pentaphyllum*.^3^ However, the immunomodulatory mechanism and effect of Gps on morphine-induced immunosuppression are still unknown. We aimed to investigate the pharmacological effects of Gps in morphine-induced immunosuppressive mice and the underlying mechanism involved.

To investigate whether Gps could reduce the incidence of MRSA pneumonia in morphine-induced immunosuppression, the mice exposed to morphine for six days were treated with Gps (120 mg/kg) and then were infected with MRSA intranasally (Fig. 1A). The six-day morphine exposure could cause weight loss of the mice (Fig. 1B-i); however, the effect could be reversed by Gps treatment (Fig. 1B-ii). Especially, Gps treatment prevented excessive weight loss after three days of MRSA infection (Fig. 1B-iii). In addition, the model group mice had a considerably higher body temperature during the infection period, which did not present in the Gps-treated mice (Fig. 1C). To verify whether Gps treatment could ameliorate the immune function in MRSA pneumonia mice, we examined the immune organ index. The data showed that the thymus index of the other three groups decreased compared with the blank group, which indicates that the thymus gland of the mice had shrunk to some extent, but the shrinkage of the Gps-treated mice was less than that of the model group mice (Fig. 1D-i, ii). Likewise, the Gps group mice also showed a significant improvement in lung coefficients compared with model group mice, which reflected the degree of pulmonary edema (Fig. 1D-iii). In addition to immune organ indexes and lung coefficient, the levels of leukocytes, lymphocytes, and neutrophils can also reflect the inflammation degree in the organism, which are considered direct indicators of inflammatory infection. In this study, the whole blood cell analysis showed a significant increase in white blood cells and neutrophils and a significant decrease in lymphocytes in the peripheral blood of the model group mice, but the expression levels of the three in the Gps group mice were consistent with those in the control and blank groups (Fig. 1E). Then, we used micro-CT and hematoxylin-eosin (H&E) staining to further evaluate the lung inflammation status. The results showed that abnormal density shadow areas (Fig. 1G) and obvious histopathological changes such as blurred and incomplete structures and a large number of inflammatory cells infiltrating the tubular lumen (Fig. 1H) were observed in the lungs of the model group mice, while the lung pathology of the Gps group mice was similar to that of the control mice. We collected mouse bronchoalveolar lavage fluid and figure out the protein expression levels of albumin and proinflammatory factors by ELISA. Compared with the control group, the expression of IL-6, IL-1β, TNF-α, and albumin significantly increased in the model group mice, while the Gps treatment significantly inhibited these increases (Fig. 1H). We also examined the transcription levels of relevant pro-inflammatory factors in mouse lung homogenates with qPCR. Compared with the control group, the expression of IL-6, IL-1β, and TNF-α dramatically elevated in the model group mice, but the expression was significantly suppressed in the Gps-treated mice (Fig. 1I). After Gps treatment, the lung bacterial load of the mice was significantly reduced compared with the model group mice (Fig. 1J).

**Figure 1:**
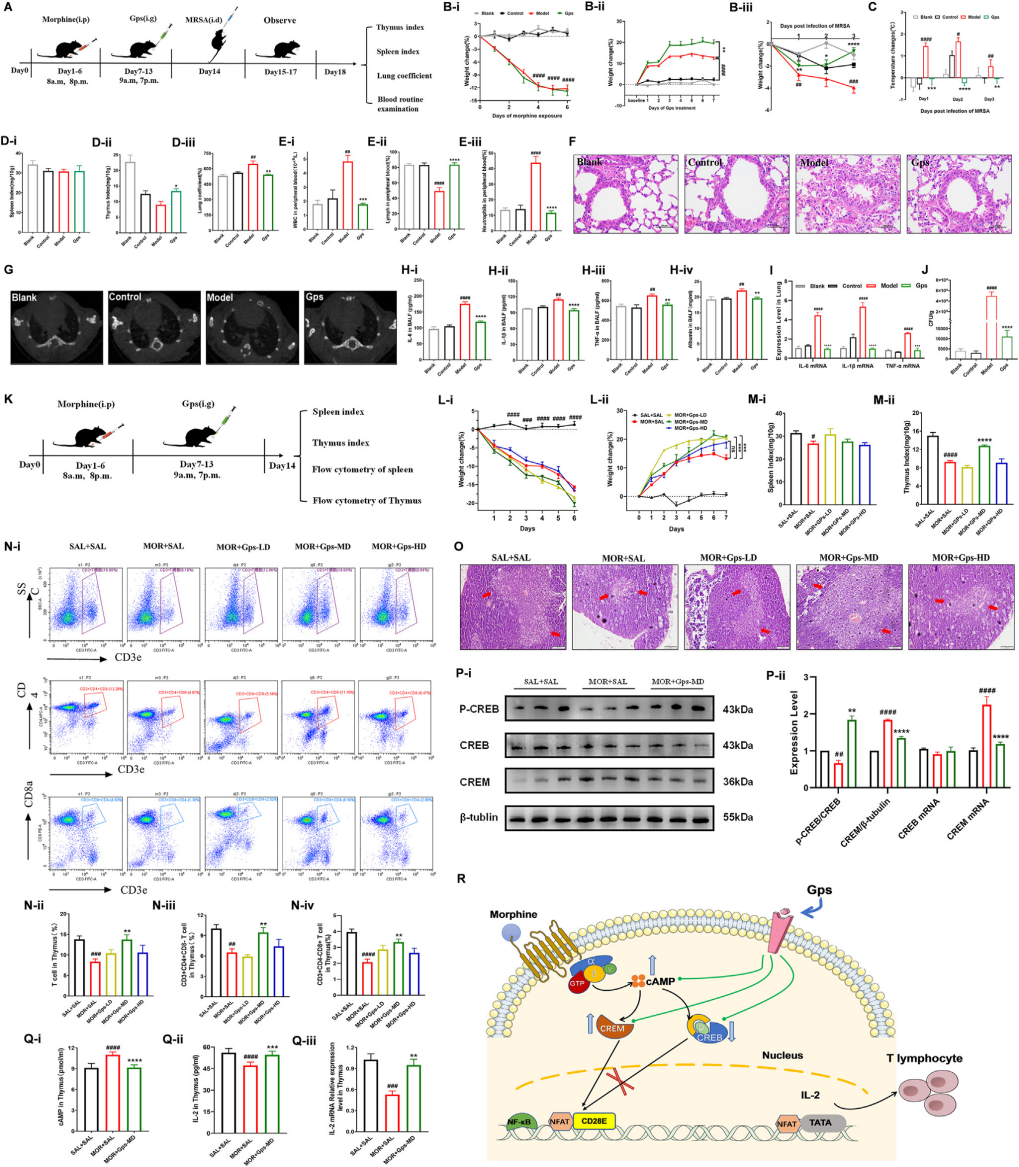
Gypenosides (Gps) are involved in regulating the proportion of thymic T lymphocyte subsets via the cAMP-CREM/CREB-IL-2 pathway in morphine-induced immunosuppression. **(A)** Schematic diagram of the infection process of MRSA after Gps treatment of morphine immunosuppression. Mice were randomly divided into four groups (10–12 mice in each group): (I) saline + saline + PBS group (Blank); (II) saline + saline + MRSA group (Control); (III) morphine + saline + MRSA group (Model); (IV) morphine + Gps + MRSA group (Gps). The mice's weight changed **(B-i)** during the morphine immunosuppression modeling, **(B-ii)** during the Gps treatment, and **(B-iii)** after infection with MRSA. **(C)** The mice's body temperature changed after infection with MRSA (*n* = 10–12). **(D-i)** Spleen index, **(D-ii)** thymus index, and **(D-iii)** lung coefficients of mice after MRSA infection. The numbers of **(E-i)** white blood cells (WBC), **(E-ii)** lymphocytes, and **(E-iii)** neutrophils in the peripheral blood of mice after MRSA infection. **(F)** Representative H&E staining of lung tissues. Original magnification: × 200. **(G)** Schematic diagram of micro-CT scan of the lungs of mice after MRSA infection. Protein expression levels of **(H-i)** IL-6, **(H-ii)** IL-1β, **(H-iii)** TNF-α, and **(H-iv)** albumin in the bronchoalveolar lavage fluid (BALF) of mice after MRSA infection. The data were presented as mean ± SEM; one-way ANOVA was applied to test for group differences (*n* = 4). **(I)** Protein expression levels of IL-6, IL-1β, TNF-α, and albumin in the BALF of mice after MRSA infection (*n* = 4). **(J)** Bacterial burden in the lung. **(K)** Schematic diagram of the experimental flow of Gps treatment for morphine immunosuppression. The mice's weight changed **(L-i)** during the morphine exposure and **(L-ii)** during the Gps treatment (*n* = 6). **(M-i)** Spleen index and **(M-ii)** thymus index of mice treated with Gps after morphine immunosuppression (*n* = 5). **(N-i)** Schematic diagram of flow cytometric analysis of the proportion of thymic T lymphocytes and their subsets in mice. **(N-ii)** Statistical analysis of the percentage of thymus T lymphocytes. **(N-iii)** Statistical analysis of the percentage of CD3^+^ CD4^+^ CD8^-^ T lymphocytes in the thymus of mice (^##^*P* < 0.01 *vs*. SAL + SAL group, ^∗∗^*P* < 0.01 *vs*. MOR + SAL group). **(N-iv)** Statistical analysis of the percentage of CD3^+^ CD8^+^ CD4^-^ T lymphocytes in the thymus of mice (*n* = 5). **(O)** Thymus sections stained with H&E and representative images (20× magnification) are shown (*n* = 4). **(P-i)** The representative immunoblots. **(P-ii)** Relative expression levels of p-CREB to CREB and CREM to β-tubulin in the thymus (*n* = 3). Relative expression levels of CREB and CREM mRNA in the thymus (*n* = 6). **(Q-i)** cAMP levels in the thymus. **(Q-ii)** Protein and **(Q-iii)** mRNA expression levels of IL-2 in the thymus (*n* = 5). **(R)** The mechanisms by which Gps ameliorates morphine-induced immunosuppression. All data were presented as mean ± SEM; one-way ANOVA was applied to test for group differences, except where specifically mentioned. Pound keys represent statistical difference from control or SAL + SAL group and asterisks represent statistical difference from model or MOR + SAL group (^####^*P* < 0.0001, ^###^*P* < 0.001, ^##^*P* < 0.01, ^#^*P* < 0.05; ^∗∗∗∗^*P* < 0.0001, ^∗∗∗^*P* < 0.001, ^∗∗^*P* < 0.01, ^∗^*P* < 0.05).

To figure out whether this effect was achieved by improving mouse immune function, we conducted further studies and screened the Gps dose by the administration at a low dose of 60 mg/kg (MOR + Gps-LD) and a high dose of 180 mg/kg (MOR + Gps-HD) to investigate its optimal dose (Fig. 1K). During the morphine treatment, the mouse body weight of the other four groups decreased significantly compared with the SAL + SAL group (Fig. 1L-i). After Gps treatment, the MOR + Gps-MD group mice and the MOR + Gps-LD group mice had rapid weight gain (Fig. 1L-ii), which was significantly different from the model group. The organ index results showed that the spleen and thymus indexes were decreased in the MOR + SAL group compared with the SAL + SAL group, but a medium dose of Gps (120 mg/kg; MOR + Gps-MD) treatment restored the decreased thymus index (Fig. 1M). Considering that the thymus is mainly composed of T lymphocytes, we detected T lymphocytes and their subsets in the thymus. The results of flow cytometry showed that the CD3^+^ T lymphocytes, CD3^+^ CD4^+^ CD8^-^ T lymphocyte subsets, and CD3^+^ CD8^+^ CD4^-^ T lymphocyte subsets were significantly decreased in the thymus of MOR + SAL group mice, whereas thymus T lymphocytes and their subsets were increased after a low-dose Gps treatment (Fig. 1N). Moreover, the results of H&E staining of the thymus showed that the medullary part of the thymus, the lymphocyte production, was significantly reduced in the MOR + SAL group compared with the control group. The medullary fraction of the MOR + Gps-MD group was similar to that of the control group (Fig. 1O).

Morphine can activate μ-opioid receptors and increase the expression of the second messenger cAMP, which promotes the expression of cAMP-responsive element modulator (CREM) and inhibits the phosphorylation level of cAMP response element binding protein (CREB). CREB binds to the IL-2 promoter to activate IL-2, while CREM competes with CREB for the binding site of the IL-2 promoter to inhibit IL-2 transcription.^4^ IL-2 is closely related to the growth, proliferation, and differentiation of thymic T lymphocytes.^5^ The ELISA results showed that Gps could significantly reduce the morphine-induced cAMP increase (Fig. 1Q-i). Meanwhile, the IL-2 level in the thymus region was also evaluated. These results presented that IL-2 protein and mRNA levels were significantly lower in the MOR + SAL group than in the SAL + SAL group, while the levels were much higher after Gps treatment (Fig. 1Q-ii, iii). We also measured the protein levels of the thymic cAMP family transcription factors CREM and CREB. The data showed that six-day morphine exposure decreased the phosphorylation level of CREB and increased CREM expression compared with the control group. Notably, Gps significantly decreased CREM expression and significantly increased the phosphorylation level of CREB (Fig. 1P-i, ii). The results of transcription factor expression were consistent with the translation level (Fig. 1P-ii).

In conclusion, our study showed that Gps could increase the proportion of thymic T lymphocytes and their subsets to reduce the risk of MRSA pneumonia after morphine-induced immunosuppression, which mainly affects the cAMP-CREM/CREB-IL-2 pathway. These findings are important for identifying new therapeutic strategies and developing more effective drugs to improve immunosuppression caused by chronic opioid treatment and reduce susceptibility to opportunistic diseases.

## Conflict of interests

The authors have no conflict of interests to declare.

## Funding

This work was supported by the 10.13039/501100001809National Natural Science Foundation of China (No. 82174498, 82174141), the National Administration of Traditional Chinese Medicine Youth Qihuang Scholars Support Project, the Open Project of Chinese Materia Medica First-Class Discipline of 10.13039/501100007956Nanjing University of Chinese Medicine (China) (No. 2020YLXK013), and the Postgraduate Research & Practice Innovation Program of 10.13039/501100002949Jiangsu Province, China (No. KYCX21_1796).
