# Supplementary material for: Gypenosides ameliorate morphine-induced immunosuppression with an increased proportion of thymic T lymphocyte subsets and are involved in the regulation of the cAMP-CREM/CREB-IL-2 pathway
Source: Genes Dis. 2023 Jul 28;11(3):101049. doi: 10.1016/j.gendis.2023.05.026 (PMC10825300; doi:10.1016/j.gendis.2023.05.026)
Supplement: Multimedia component 1 [file mmc1.docx]

**MATERIALS AND METHODS**

**Reagents**

Morphine injection from Northeast Pharmaceutical Group Shenyang No. 1 Pharmaceutical Co., Ltd (Lot No. 201013, Shenyang, China); Gypenoside purchased from Shanghai Yuanye Biological (Lot No. S31402, Shanghai, China); MRSA (Category: USA300, Gift of Prof. Liyun Shi’s Lab).

**Animals**

The 8-10-week-old male C57BL/6 mice utilized for this investigation were supplied by Jiangsu Changzhou Kavins Laboratory Animal Co., Ltd. (Animal Certification Number: SCXK (SU)2016-0010). All mice were housed in a Special Pathogen-Free Environment. All mice had free access to food and water, and the temperature was 22°C, the relative humidity was 45%-55%, and the ventilation was clean, the light/dark cycle is 12 hours per day. All laboratory procedures are conducted in compliance with National Institutes of Health and Nanjing University of Chinese Medicine guidelines (Animal Code of Ethics: 202104A010).

**Mouse model of bacterial pneumonia caused by morphine abuse**

Referring to the classical method of establishing the morphine immunosuppressive model in the literature,^35^ the dose of morphine was selected, and the morphine abuse immunosuppressive model was treated with an intraperitoneal incremental injection of morphine. In short, after morphine treatment, Gps treatment was followed by nasal infection of MRSA, as shown in Fig. 1A. Mice were arbitrarily divided into four groups (10-12 mice in each group): (I) saline+saline+PBS group (blank); (II) saline+saline+MRSA group (control); (III) morphine+saline+MRSA group (model); (IV) morphine+Gps+MRSA group (Gps). Morphine dose was progressively increased as follows: day 1, 10 mg/kg; day 2, 20 mg/kg; day 3, 40 mg/kg; day 4, 60 mg/kg; day 5, 80 mg/kg; day 6, 100 mg/kg; twice a day. On the seventh day, the Gps group mice were administrated with Gps (120 mg/kg) by gavage twice a day for 7 consecutive days, whereas other groups mice were given PBS. Thereafter, 50L of regular PBS or MRSA (1×10^9^ CFU/mouse) was administered by nasal drip. Mice were sacrificed three days after infection. The body weight and anal temperature were recorded every day. The spleen, thymus and lung tissues of mice were removed and weighed.

**Micro-computed tomography**

All specific use methods and details of the dedicated whole-body small animal micro-CT scanner were prepared as described previously (SkyScan 1176, Bruker micro-CT, Kontich, Belgium)^36^.

**Complete blood cell analysis**

Pure white blood cells and leukocyte subsets (eosinophils, neutrophils, lymphocytes, and monocytes) were counted using the Automatic Hematology Analyzer (Mindray, BC2800Vet).

**Albumin analyses**

All specific use methods and details were prepared as described previously (ML-E-24109, Dongguan Enzyme Link Biotechnology Co., Ltd., China) according to the manufacturer’s instructions.

**Enzyme-linked immunosorbent assay (ELISA)**

The levels of cAMP and IL-2 in Thymus and TNF-α, IL-1β and IL-6 in BALF were measured with commercial ELISA kits according to the manufacturer's instructions. These kits are from Shanghai Enzyme Link and Dongguan Enzyme Link Biotechnology Co., Ltd. Absorbance was determined using an ELx800 Universal Microplate Reader (BIO-TEK, Vermont, USA).

**Quantitative Real-time PCR**

Specific operation and details of RNA extraction and determination of RNA concentration and purity have been outlined as previously published. 37 Then, real-time PCR was conducted using SYBR@ Green (YESEN, China, H1211170) following the manufacturer’s instructions. The primer sequences were shown in Fig.S1.

**Histologic and immunohistochemical analyses**

The Thymus and lung tissue samples were collected and rinsed with ice cold PBS, fixed in 4% paraformaldehyde (Biosharp, China) for 24 h, embedded in paraffin and stained with hematoxylin and eosin (H&E) (Beyotime, China).

**Flow cytometry**

According to the previous results, in order to further explore the improvement effect of Gps on immune function of mice, we further studied the dose of Gps. After immunosuppression modeling, different doses of Gps were selected for treatment, and the changes of immune organs of mice were observed, as shown in Fig. 4A. The grouping of mice is as follows (4-6 mice in each group): (I) saline+saline group (SAL+SAL); (II) morphine+saline group (MOR+SAL); (III) morphine+Gps 60 mg/kg group (MOR+Gps LD); (IV) morphine+Gps 120 mg/kg group (MOR+Gps MD); (v) Morphine+Gps 180 mg/kg group (MOR+Gps HD). After Gps treatment, the spleen and thymus of mice were collected, and their organ indexes were recorded. Then, single cell suspension was prepared and treated with erythrocyte lysate (143191, Biosharp, China). Add the corresponding fluorescent labeled antibody (Anti-CD3e-FITC, eBioscience, 11-0031-82; Anti-CD4-APC, eBioscience, 17-0041-81; Anti-CD8a-PE, eBioscience, 12-0081-81; Anti-CD11b-FITC, eBioscience, 11-0112-82; Anti-ly-6G-APC, eBioscience, 17-9668-82; Anti-CD19-APC, eBioscience, 17-0193-80) to the washed cell suspension according to the dosage specified in the instructions, shake evenly, and incubate on ice at 4℃ in the dark for 40 min. After incubation, the cells were washed and detected by flow cytometry (CytoFLEX, Beckman Coulter, USA). The experimental results were obtained by CytExpert 2.3 Analysis results.

**Western Blot**

Total protein was extracted using RIPA lysis buffer (P0013C, Beyotime, China). The proteins from thymus tissue were separated in 10% g SDS-PAGE and transferred to PVDF membrane (Millipore, USA). The membranes were blocked for one hour at room temperature in TBST containing 0.1%Tween-20 and 5%BSA. After blocking, the membranes were treated with primary antibodies for CREM (1:1000 dilution, 12131-1-AP, Proteintech), p-CREB (1:1000 dilution, # 9198S, Cell Signaling Technology), CREB (1:1000 dilution, #9197S, Cell Signaling Technology) or β-tubulin (1:1000 dilution, #2128S, Cell Signaling Technology) overnight at 4℃. After washing with TBST buffer, the membrane was incubated with HRP-conjugated anti-rabbit and anti-mouse at room temperature for 1 h. After washing with TBST buffer, the signals were detected by ECL reagent (Millipore, WBKLS0100, United States) and then measured by gel imaging system (Tanon, China) and Tanon images were used for quantification. Supplementary materials include full-length original imprinted or gel as shown in Fig.S2.

**Statistical Analysis**

All mice were randomized and assigned to groups. All analysis were performed using GraphPad Prism 8.0. Statistical detection methods include student’s unpaired t-test, one-way ANOVA analysis with post hoc Bonferroni test. Statistical significance was defined as a probability value less than 0.05.
